# Curcumin Attenuates Periodontal Injury via Inhibiting Ferroptosis of Ligature-Induced Periodontitis in Mice

**DOI:** 10.3390/ijms24129835

**Published:** 2023-06-07

**Authors:** Yawei Wang, Hongbing Lin, Wenxin Huang, Zixian Liu, Zhen Chen, Xuetao Zhao, Tong Ding, Wenguang Qin, Yuqin Shen

**Affiliations:** 1 Department of Periodontics, Affiliated Stomatology Hospital of Guangzhou Medical University, Guangdong Engineering Research Center of Oral Restoration and Reconstruction, Guangzhou Key Laboratory of Basic and Applied Research of Oral Regenerative Medicine, Guangzhou 510182, China; 2 Jilin Provincial Key Laboratory of Tooth Development and Bone Remodeling, Hospital of Stomatology, Jilin University, Changchun 130021, China

**Keywords:** curcumin, periodontitis, ferroptosis, SLC7A11, GPX4

## Abstract

Periodontitis is a chronic infectious disease characterized by the destruction of connective tissue and alveolar bone that eventually leads to tooth loss. Ferroptosis is an iron-dependent regulated cell death and is involved in ligature-induced periodontitis in vivo. Studies have demonstrated that curcumin has a potential therapeutic effect on periodontitis, but the mechanism is still unclear. The purpose of this study was to investigate the protective effects of curcumin on alleviating ferroptosis in periodontitis. Ligature-induced periodontal-diseased mice were used to detect the protective effect of curcumin. The level of superoxide dismutase (SOD), malondialdehyde (MDA) and total glutathione (GSH) in gingiva and alveolar bone were assayed. Furthermore, the mRNA expression levels of *acsl4*, *slc7a11*, *gpx4* and *tfr1* were measured using qPCR and the protein expression of ACSL4, SLC7A11, GPX4 and TfR1 were investigated by Western blot and immunocytochemistry (IHC). Curcumin reduced the level of MDA and increased the level of GSH. Additionally, curcumin was proven to significantly increase the expression levels of SLC7A11 and GPX4 and inhibit the expression of ACSL4 and TfR1. In conclusion, curcumin plays a protective role by inhibiting ferroptosis in ligature-induced periodontal-diseased mice.

## 1. Introduction

Periodontitis is an inflammatory disease of tooth-supporting structures, which leads to tissue destruction and tooth loss as a result of the interaction of dental plaque microorganisms and the host’s immune response [1]. In recent years, the importance of increased oxidative stress was highlighted as a potential risk factor in the course of periodontal disease [2,3]. In periodontitis pathogenesis, once activated by subgingival microbes (*P. gingivalis*) and their virulence factors (LPS) [4], macrophages can not only engulf periodontal pathogens but also release reactive oxygen species (ROS), contributing to the onset of periodontitis as well as the activation of acquired immunity [5,6]. The generation of ROS can be considered as a “double-edged” sword because physiological ROS plays an important role in cell signaling, gene regulation and antimicrobial defense [7], but an overabundance results in oxidative stress which can lead to irreversible tissue injury [8,9]. During periodontitis, ROS (especially O^2−^, H_2_O_2_ and OH^•^) acts mainly through lipid peroxidation [10], and leads to an oxidative imbalance that triggers proinflammatory mechanisms, and importantly osteoclast genesis, which then leads to the bone loss [11,12]. Some studies have implied the levels of lipid peroxidation in salivary and plasma of periodontitis patients were increased compared with those in 20 healthy controls [13], and lipid peroxidation may contribute to the degradation of cellular homeostasis and ultimately result in cell death [14].

Ferroptosis is a newly identified form of programmed cell death characterized by the accumulation of lipid hydroperoxides, which is induced by the exposure of lipids to ROS [15]. The biochemical characteristics of ferroptosis include ROS accumulation, mitochondrial shrinkage, glutathione (GSH) depletion, glutathione peroxidase 4 (GPX4) antioxidant dysfunction and lipid peroxide accumulation [16]. Recent studies revealed that ferroptosis plays a regulatory role in inflammatory diseases, including periodontitis [17,18]. For example, lipopolysaccharide from *P. gingivalis* (Pg-LPS), one major putative pathogen for periodontitis, may elicit high ROS production, mitochondrial dysfunction and lipid peroxidation [19]. Ferroptosis in periodontal ligament stem cells promotes the development of periodontitis [20]. These findings strongly suggest that the treatments targeting ferroptosis could be potential strategies in the treatment of periodontitis.

Curcumin is a main active ingredient of the Chinese herbal medicine Curcuma longa which possesses strong pharmacological properties such as antioxidant, antiapoptotic and neuroprotective effects in various diseases [21,22,23]. It has been reported that excessive ROS production and lipid peroxidation could be suppressed by curcumin intervention [24]. It is likely that curcumin modulates ferroptosis by decreasing oxidative stress and its consequences, such as lipid peroxidation [25]. For example, curcumin can protect against renal damage in rhabdomyolysis-AKI via attenuating the characteristic changes of lipid peroxidation and GSH depletion in ferroptosis [26]. Curcumin may play a crucial role in the treatment of periodontitis, and this role may be decided by the strong antioxidant capacity of curcumin related to scavenging excessive ROS [27,28]. In animal models of periodontitis, curcumin down-regulated the inflammatory cytokines interleukin-1β (IL-1β), interleukin-6 (IL-6) and tumor necrosis factor-α (TNF-α) and suppressed bone resorption by the suppression of RANKL expression [29,30]. However, whether curcumin exerts protective effects against periodontitis via reducing ferroptosis is still unclear.

Therefore, the objective of this study was to investigate whether curcumin could attenuate periodontal injury via inhibiting ferroptosis of ligature-induced periodontitis in mice. The findings are expected to provide a novel therapeutic target for patients with periodontitis.

## 2. Results

The placement of a ligature between the maxillary first molars and the second molars teeth mimics the development of the human periodontal disease, leading to bacterial plaque accumulation, local cellular inflammatory induction, damage to gingival tissue and eventually bone loss. The establishment of correct and reasonable animal models is conducive to deeply study the key factors affecting the occurrence and development of periodontitis, and provides ideas for disease control and drug research.

### 2.1. Curcumin Attenuates Periodontal Injury in Gingival Tissue of Ligature-Induced Periodontitis in Mice

**Figure 1:**
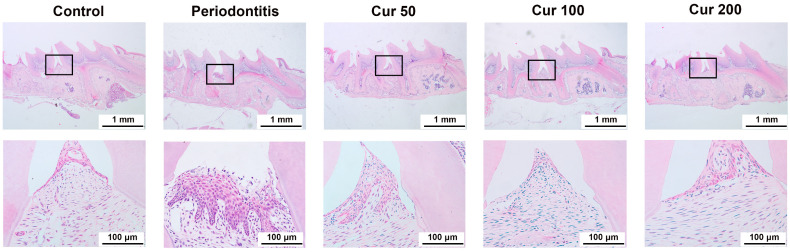
Curcumin attenuates periodontal injury in gingival tissue of ligature-induced periodontitis in mice. H&E staining of gingival tissue.

H&E staining (Figure 1) showed pathological changes in the gingival tissue region, with the substantial infiltration of inflammatory cells and a disorganized connective tissue structure in the periodontal tissues of the periodontitis group, indicating an extensive inflammatory state. On the contrary, inflammatory cell infiltration was significantly reduced after treatment with different concentrations of curcumin, especially in the Cur 100 group, which indicates that curcumin plays an important role in protecting periodontal injury in the gingival tissue of ligature-induced periodontitis in mice.

### 2.2. Curcumin Inhibits Ferroptosis-Related Marker in Gingival Tissue of Ligature-Induced Periodontitis in Mice

**Figure 2:**
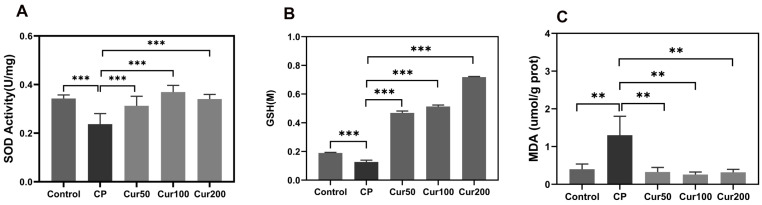
Curcumin influences ferroptosis-related molecular marker in gingival tissue of ligature-induced periodontitis in mice. (**A**) Detection of SOD level in plasma. (**B**) Detection of MDA level in gingiva tissue. (**C**) Detection of GSH level in gingiva tissue. ** *p*  <  0.01 compared to LPS group; *** *p*  <  0.001 compared to LPS group.

Superoxide dismutase (SOD) is a principal antioxidant enzyme that protects cells from oxidative damage by converting superoxide anion radicals to hydrogen peroxide and oxygen in mitochondria [31]. As shown in Figure 2A, the average SOD activity in the plasma of mice in the control group was 0.324 U/mg, and the SOD activity in the plasma of mice in the CP group was significantly lower compared to the control group (0.237 U/mg vs. 0.324 U/mg). After different doses of curcumin intervention, SOD activity in the plasma of mice in Cur 50, Cur 100 and Cur 200 was significantly higher than that in CP (0.312 U/mg vs. 0.237 U/mg; 0.369 U/mg vs. 0.237 U/mg; 0.340 U/mg vs. 0.237 U/mg). The curcumin groups showed significantly increased SOD activity in the plasma of mice compared with the periodontitis group when oral bacteria accumulated in the ligature for 10 d (*p* < 0.01).

GSH is a crucial intracellular non-enzymatic antioxidant, which exists mostly in the cytoplasm and in some certain organelles (such as mitochondria) and act as a substrate for GPX4-mediated lipid detoxification [32]. GSH depletion caused by cysteine deprivation directly inactivates GPX4 and leads to the subsequent induction of ferroptosis [33]. The GSH content of gingival tissue in the CP group was significantly lower than that in the control group (0.126 M vs. 0.189 M). After treatment with curcumin, the content of GSH in the gingival tissue of mice was significantly increased (0.467 M vs. 0.126 M; 0.513 M vs. 0.126 M;0.718 M vs.0.126 M), indicating that curcumin has the effect of increasing the GSH content within gingival tissue. (Figure 2B).

Lipid peroxidation is a prevalent feature of a few disease states, which mediated membrane damage which is dominant to ferroptosis [34]. The accumulation of MDA in the gingival tissue in the CP group was significantly higher than that in the control group (1.299 umol/g prot vs. 0.399 umol/g prot). The content of MDA in the gingival tissue of mice in the curcumin group was significantly decreased (0.326 umol/g prot vs. 1.299 umol/g prot; 0.258 umol/g prot vs. 1.299 umol/g prot; 0.317 umol/g prot vs. 1.299 umol/g prot), indicating that MDA content in the gingiva tissue of the mice in the periodontitis group was significantly higher than that in the control group. The curcumin administration significantly reduced MDA levels in the periodontitis group (Figure 2C).

### 2.3. Curcumin Regulates Ferroptosis-Related Genes and Proteins in Gingival Tissue of Ligature-Induced Periodontitis Mice

**Figure 3:**
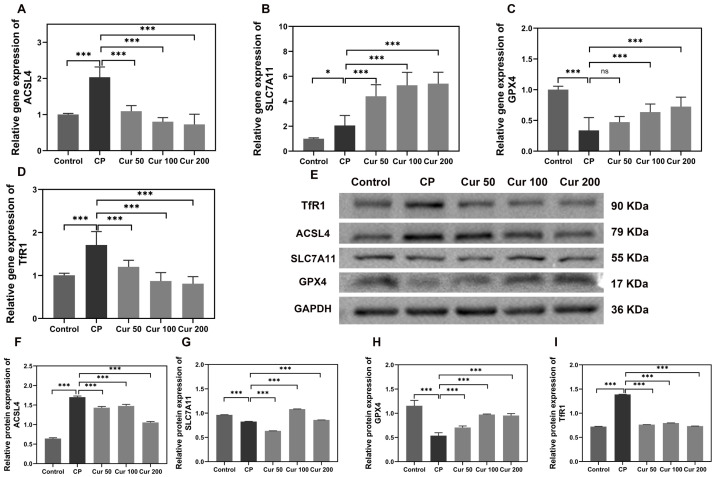
Curcumin regulates the expression of ferroptosis-related genes and proteins in gingival tissue. (**A**–**D**) qPCR analysis of the mRNA content of ferroptosis gene in gingiva. (**E**–**I**) Western blot detection of the changes in ferroptosis protein in gingiva. “ns” means no significant difference (*p*  >  0.05); * *p*  <  0.05 compared to LPS group; *** *p*  <  0.001 compared to LPS group.

Additionally, the changes in ferroptosis-related genes and proteins (ACSL4, SLC7A11, GPX4 and TfR1) were additionally detected by qPCR and Western blot. ACSL4 is an important enzyme involved in lipid metabolism and TfR1 imports iron from the extracellular environment into cells, contributing to the cellular iron pool required for ferroptosis [35,36]. The expression levels of ACSL4 and TfR1 were increased in the periodontitis group, and curcumin inhibited the expression of them (Figure 3A,D–F). SLC7A11 is a cystine/glutamate antiporter with critical functions of importing extracellular cysteine for GSH biosynthesis and GPX4 is a lipid repair enzyme, which represses ferroptosis by reducing lipid ROS [37]. Silk ligation significantly decreased the expression of SLC7A11 and GPX4 compared with the control group. However, the treatment with curcumin significantly increased the levels of SLC7A11 and GPX4 (Figure 3A–D).

### 2.4. Curcumin Regulates Ferroptosis-Related Proteins in Gingival Tissue Detected by Immunohistochemistry (IHC)

To further investigate the occurrence of ferroptosis in periodontitis, we tested the expression level of ferroptosis-markers such as ACSL4, SLC7A11, GPX4 and TfR1 in the gingival tissue. The IHC staining demonstrated a significantly decreased GPX4 and SLC7A11 expression in the periodontitis and Cur 50 group compared with the control group and the other curcumin group (Figure 3A,B). In ligature-induced periodontitis in mice, the expression level of ACSL4 and TfR1 were significantly increased, which would induce the initiation of ferroptosis, the while curcumin treatment can decrease the expression of those proteins (Figure 3C,D). The IHC staining results were consistent with those of qPCR and Western blotting. These results indicated that ferroptosis plays a regulatory role in ligature-induced periodontitis, and curcumin could, through inhibiting ferroptosis, alleviate ligature-induced periodontal injury.

### 2.5. Curcumin Inhibits Ferroptosis-Related Makers and Genes in Alveolar Bone of Ligature-Induced Periodontitis in Mice

**Figure 4:**
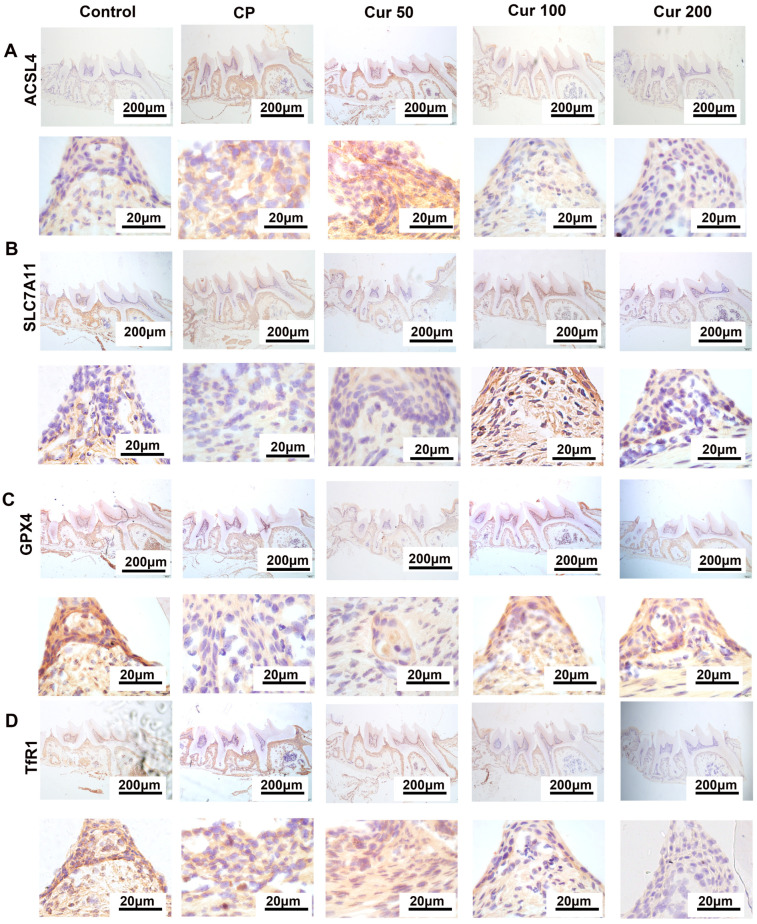
IHC detection of the changes in ferroptosis protein markers in gingiva. (**A**) ACSL4 expression detected by IHC staining in gingiva. (**B**) SLC7A11 expression detected by IHC staining in gingiva. (**C**) GPX4 expression detected by IHC staining in gingiva. (**D**) TfR1 expression detected by IHC staining in gingiva.

Periodontitis is a typical chronic inflammatory disease that is associated with not only gingival inflammation but also alveolar bone loss. The occurrence of bone resorption indicates that homeostasis in periodontal tissue is absent under inflammatory conditions [38].The treatment effect of curcumin in periodontal disease includes anti-inflammation and relieves alveolar bone resorption. Therefore, we have further detected ferroptosis related markers in alveolar bone tissues. Our results found that ligature-induced periodontitis in mice increased alveolar bone tissue MDA content levels and was accompanied by a decrease in the levels of alveolar bone tissue GSH when compared with the control group. However, the results in the curcumin groups revealed a decrease in MDA, and increase in GSH (Figure 4A,B). These results were consistent with gingival tissue, indicating that curcumin can decrease the expression level of ferroptosis-related molecular markers. 

**Figure 5:**
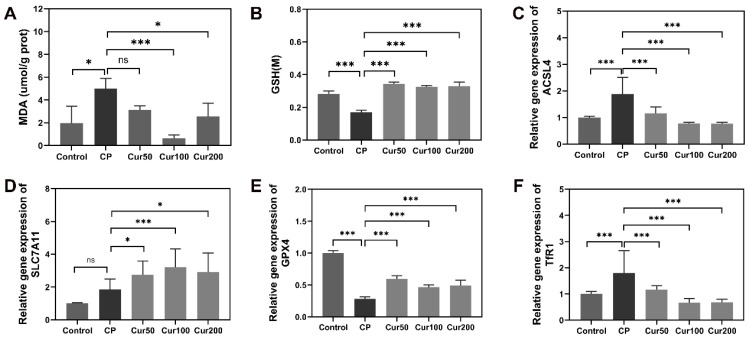
Curcumin attenuates ferroptosis in alveolar bone of ligature-induced periodontitis in mice. (**A**) Detection of MDA level in alveolar bone tissue. (**B**) Detection of GSH level in alveolar bone tissue. (**C**–**F**) qPCR analysis of the mRNA of ferroptosis markers in alveolar bone. “ns” means no significant difference (*p*  >  0.05); * *p*  <  0.05 compared to LPS group; *** *p*  <  0.001 compared to LPS group.

Furthermore, we measured the expression of *acsl4*, *slc7a11*, *gpx4 and tfr1* in the alveolar bone by qPCR. The results showed that scl7a11 and gpx4 expression decreased and the level of acsl4 and tfr1 were increased in periodontitis group; however, the expression of slc7a11 and gpx4 were increased in the curcumin groups and curcumin can decrease the expression of acsl4 and tfr1 (Figure 5C–F). These findings further confirmed that lipid peroxidation and ferroptosis are involved in ligature-induced periodontal disease, and that curcumin can regulate ferroptosis-related markers to ameliorate alveolar bone resorption.

## 3. Discussion

Periodontitis is an infectious disease caused by periodontal pathogenic bacteria and is one of the chronic diseases with the highest incidence in the oral cavity, which makes it an important disease of great concern in the field of public health. The ligature-induced periodontitis mice model is a classical experimental model to mimic periodontitis in vivo. Although the exact mechanism of periodontitis pathogenesis is not fully elucidated, many studies have shown that it is linked to ROS-mediated oxidative stress [39]. Curcumin is a potent antioxidant that facilitates mitochondrial activities, reduces the level of ROS and lipid peroxidation, improves SOD activity and inhibits erastin-induced ferroptosis [40,41]. Many studies have shown that curcumin has a potential role in the treatment of periodontitis [42,43]. However, the exact mechanism of curcumin in ligature-induced periodontitis has not been studied clearly. 

Curcumin is an antioxidant molecule that protects against alveolar bone resorption associated with periodontitis. However, the protective effects of curcumin in periodontitis are not fully clear. In our study, we investigated whether the protective effect of curcumin was related to a reduction in ferroptosis. In our study, we found that curcumin can exert a protective effect on gingival tissue under the circumstance of periodontitis, and significantly inhibited ferroptosis by increasing SOD activity and total GSH expression level, decreasing lipid peroxidation accumulation. Moreover, ligature-induced periodontitis in mice treated with curcumin exhibited increased expression levels of SLC7A11 and GPX4 and decreased expression levels of ACSL4 and TfR1. Our study demonstrated that curcumin can protect against periodontitis by inhibiting ferroptosis. We have presented evidence suggesting that ferroptosis is involved in the development and progression of ligature-induced periodontitis, and curcumin attenuates ferroptosis by decreasing the ROS and MDA accumulation and reversing the down-regulation of GPX4 and SLC7A11.

Oxidative stress is a phenomenon caused by an imbalance between the production and accumulation of ROS in cells and tissues and the ability of a biological system to detoxify these reactive products [44]. Oxidative stress is one of the main reasons for the inefficient repair of periodontitis by those clinical treatments [45]. MDA is a crucial and sensitive biomarker of oxidative damage and is also a commonly used index of membrane lipid peroxidation in ferroptosis. In contrast, the antioxidant systems, including SOD and GSH, are the main factors comprising the first step of antioxidant defense mechanisms. Previous studies have confirmed that oxidative stress is a critical mechanism of ligature-induced periodontitis [3]. In our study, ligature-induced periodontitis expressed the over-production of ROS, accumulation of MDA and reduction of total GSH. Moreover, the activity of antioxidant enzymes SOD in the periodontitis group was inhibited. The curcumin pretreatment inhibited the generation of ROS and MDA, enhanced the activities of SOD and increased the levels of the antioxidants GSH. These results suggest that curcumin plays an important role in decreasing lipid peroxidation products and ROS accumulation, and ROS scavenging is one of the important mechanisms by which curcumin prevents periodontitis.

Ferroptosis is defined as an iron and lipid peroxidation dependent form of programmed cell death with typical morphological features of dysmorphic small mitochondria [15]. Importantly, increasing evidence suggests that ferroptosis could be involved in the progress of periodontitis [18]. The characteristics of ferroptosis are consistent with several hallmarks of periodontitis pathogenesis [46]. For example, MDA, a by-product of lipid peroxidation and a marker of ferroptosis, has been reported in periodontium from patients with periodontitis [13] and in periodontal tissue, polyunsaturated fatty acids (PUFAs) increase and undergo a series of peroxidation reactions driven by LOX [47]. Ferroptosis is involved in inflammatory processes in HGFs upon *P.g*-LPS stimulation and observed in the gingival tissue of periodontitis rats [19]. SLC7A11 is the light chain subunit protein and consists of the cystine/glutamate antiporter System X^c−^ [48]. The exchange of intracellular glutamate and extracellular cystine on the cell membrane is mediated by System X^c−^, which is vital to antioxidant GSH synthesis [34,49,50,51]. GPX4 is an important cofactor that detoxifies hydroperoxides in complex lipids by using GSH. The inhibition of SLC7A11 can cause ferroptosis through reducing the GSH level, and subsequently leads to the accumulation of ROS that finally results in lipid peroxidation [51]. We found that the expression of SLC7A11 and GPX4 was suppressed in the periodontitis group, and curcumin significantly improved the expression of SLC7A11 and GPX4 verified by qPCR, Western blotting and IHC assay. This suggested that curcumin may exert its protective effect on periodontitis by inhibiting ferroptosis via the SLC7A11/GPX4 axis. Moreover, ACSL4 is an important isoenzyme of polyunsaturated fatty acids (PUFAs) metabolism, which is responsible for the esterification of CoA into free fatty acids, and the formation of acyl-CoA activates the corresponding fatty acids for lipid peroxidation [52]. TfR1 is a specific ferroptosis marker, transferring iron from the extracellular environment into cells and contributing to the cellular iron pool required for ferroptosis [35]. We found that the expression of ACSL4 and TfR1 was increased in ligature-induced periodontitis in mice, and curcumin significantly decreased the expression of ACSL4 and TfR1 in ligature-induced periodontal inflammation in mice. This suggested that curcumin may exert its protective effect on ligature-induced periodontal inflammation in mice by inhibiting ferroptosis not only via SLC7A11/GPX4 but also using ACSL4 and TfR1.

Nevertheless, our study was limited by several factors. Firstly, our present study only explored curcumin’s role in lipid peroxidation and System Xc in inhibiting ferroptosis, while it could also inhibit ferroptosis through a variety of other ways. Secondly, the study only evaluated the anti-ferroptosis properties of curcumin without further investigating its effects on exact mechanisms via gene transfection or knockout. To these, further studies on the mechanisms of curcumin in inhibiting ferroptosis in periodontitis should be conducted to shed more light on the subject.

## 4. Materials and Methods

### 4.1. Reagents

Curcumin was obtained from Yuanye Biotechnology Co. (Shanghai, China). TRIeasyTM Total RNA Extraction Reagent, Hifair^®^ Ⅲ Reverse Transcriptase and Hieff^®^ qPCR SYBR Green Master Mix (No Rox) were provided by Yeasen Biotechnology Co., Ltd. (Shanghai, China). Anti-GAPDH and secondary antibodies (goat anti-rabbit and anti-mouse IgG) were purchased from Proteintech Group (Wuhan, China) and the other primary antibodies (anti-ACSL4, anti-SLC7A11, anti-GPX4 and anti-TfR1) were purchased from Abcam (Cambridge, MA, USA). The Western and IP cell lysis buffer, Total SOD assay kit, MDA detection kit and total GSH assay kit were all obtained from Beyotime Biotechnology Co. (Shanghai, China). The pepsin digestive solution, mouse enhanced polymer assay system assay kit and DAB staining solution were all obtained from ZSGB Biotechnology Co. (Beijing, China).

### 4.2. Animals Experiment

Forty-five male C57/BL mice (body weight of 20 ± 2 g) were randomly divided into five groups (for each group, *n* = 9), i.e., healthy mice without periodontitis (control), periodontitis without curcumin treatment (periodontitis) and periodontitis treated with different concentrations of curcumin. The low concentration curcumin treatment group (periodontitis + Cur 50 group), middle concentration curcumin treatment group (periodontitis + Cur 100 group) and high concentration curcumin treatment group (periodontitis + Cur 200 group) were the mice administered with 50, 100 and 200 mg·kg^−1^·d^−1^ curcumin. To establish the periodontitis model, the C57/BL mice were anesthetized using pentobarbital sodium (50 mg/kg of body weight) at first, and then a ligature wire with the diameter of 0.1 mm was placed between the maxillary first molars and the second molars [53]. The curcumin was administered to the curcumin groups (Cur 50 group, Cur 100 group and Cur 200 group) by oral gavage to the mice for 10 days. All animal experimental procedures in the present study were approved by the Animal Experimental Ethics Committee of Guangdong Huawei Testing Co., Ltd. (Guangzhou, China)(approval number: 202208003).

### 4.3. Determination of SOD in Plasma

All animals were sacrificed at 10 days after surgery. The blood was collected with an anticoagulant tube centrifuged at 4 °C 600× *g* for 10 min. The supernatant was pipetted into another new 1 mL centrifuge tube, diluted with an appropriate amount of normal saline (1:10) and then tested as a plasma sample according to the total SOD assay kit instructions.

### 4.4. Detection of Lipid Peroxidation MDA in Maxillary Tissue

Maxilla specimens were harvested and frozen into liquid nitrogen. Each group of gingiva and alveolar bone was washed briefly by 1 mL PBS then lysed in Western and IP cell lysis buffer. After centrifugation at 10,000× *g* for 10 min at 4 °C, the supernatant was collected for quantitative analysis using an MDA assay kit according to the manufacturer’s protocols. 

### 4.5. Detection of Total GSH in Maxillary Tissue

The maxilla specimens were quick-frozen with liquid nitrogen and then grinded into powder. For every 10 mg of ground tissue powder, 30 μL of protein removal reagent S solution was added and thoroughly vortexed. Then, 70 microliters of protein removal reagent S solution was added and fully homogenized with a glass homogenizer. After standing 10 min at 4 °C, the specimens were centrifuged at 10,000× *g* for 10 min at 4 °C and the supernatant was collected for the determination of total glutathione using the total glutathione assay kit according to the manufacturer’s suggestion.

### 4.6. Reverse Transcription-Quantitative (qPCR)

The total RNA of gingiva and alveolar bone was extracted according to the instructions of the TRIeasyTM Total RNA Extraction Reagent, and then the collected RNA was reverse-transcribed to cDNA with the Hifair^®^ Ⅲ Reverse Transcriptase, and further amplified by the Hieff^®^ qPCR SYBR Green Master Mix (No Rox) and finally the expression of cellular ferroptosis related-genes (acsl4, slc7a11, gpx4 and tfr1) in each group was detected by the Real-Time PCR System (LightCycler480 II, Roche, Switzerland). Cycler settings were as follows: denaturation at 95 °C (1 min), 40 amplification cycles (95 °C, 10 s) and termination (60 °C, 30 s). Quantification was carried out using the 2^−ΔΔT^ method. The sequence of primers used in the experiment is shown in Table 1.

### 4.7. Western Blot

Maxillary tissue was washed with 0.9% normal saline and then lysed in Western and IP cell lysis buffer containing a protease and phosphatase inhibitor cocktail, centrifuged at 12,000× *g* at 4 °C for 10 min. Protein concentration was quantified using the BCA assay method. Equal amounts of proteins from each sample were isolated via 10% SDS-PAGE, transferred onto PVDF membranes, and blocked with 5% milk for 1 h. After washing in 1×TBST for 1 h, the membranes were incubated with primary antibodies against ACSL4 (1:1000), SLC7A11(1:1000), TfR1(1:1000), GPX4 (1:2000) and GAPDH (1:10,000) overnight at 4 °C. After three washes with TBST, these membranes were incubated with corresponding secondary antibodies including goat anti-rabbit IgG and rabbit anti-mouse IgG for 1 h at 25 °C. Protein bands were visualized using the hypersensitive ECL chemiluminescence kit and captured using the Gel imaging system (C-Digit, Lincoln, NE, USA). The optical density of the bands was analyzed by ImageJ software V10.

### 4.8. H&E Staining

The left maxillary alveolar bones from experimental mice were taken out and fixed with 4% paraformaldehyde, placed on a shaker and then decalcified in 0.5 M EDTA for 2 weeks, followed by embedding in paraffin to obtain 3 μm thick sections on the midsagittal plane of the second molar. Sections contained teeth, gingival tissue and alveolar bone which were then stained with an HE Kit (Solarbio, China) according to the instructions, then samples were observed under a light microscope (OLYMPUS BX53F).

### 4.9. Immunohistochemistry

The expression of ACSL4 (1:500), SLC7A11 (1:200), GPX4 (1:200) and TfR1 (1:200) in the maxillae tissues was detected using immunohistochemistry (IHC) staining. Paraffin sections were dewaxed and rehydrated, and antigen repair was performed by a pepsin digest solution at 37 °C for 10 min. After recovery to room temperature, endogenous peroxidase was inactivated by the dropwise addition of an endogenous peroxidase blocker solution for 10 min and the antigen was blocked with 1% BSA (dissolved in PBS) for 60 min. The sections were incubated with a primary antibody against ACSL4, SLC7A11, GPX4 and TfR1 overnight at 4 °C. A secondary antibody coupled to horseradish peroxidase-conjugated goat anti-rabbit IgG (1:100 dilution) was added dropwise and incubated for 60 min at 37 °C. A DAB chromogenic solution was used for color development. Hematoxylin restaining was followed by alcohol gradient dehydration. The slices were sealed with a neutral gum after clearing in xylene and observed at 100× magnification by light microscopy (OLYMPUS BX53F).

### 4.10. Statistical Analysis

All statistical analyses were performed using the GraphPad Prism version 8.0 software (GraphPad 8.0, San Diego, CA, USA). Data are presented as mean ± standard deviation. *p*-values are calculated with a one-way analysis of variance. A *p*-value less than 0.05 was considered statistically significant.

## 5. Conclusions

In conclusion, our study show that curcumin can alleviate ligature-induced periodontitis by inhibiting ferroptosis. Further, it was evident that curcumin is an effective therapeutic strategy to rescue the gingiva and alveolar bone from inflammation induced by an accumulation of oral microorganisms through its anti-ferroptosis activity. Therefore, our findings suggested another regulatory mechanism of curcumin treatment in periodontitis, which may provide a developing therapy for periodontitis treatment and hint at more effective methods in targeting ferroptosis for the treatment of periodontitis.

## Figures and Tables

**Table 1:** Primer Sequence.

Primer	Forward Primer Sequence (5′-3′)	Reverse Primer Sequence (5′-3′)
*gapdh*	GGAGATTGTTGCCATCAACGA	GAAGACACCATAGACTCCACG
*acsl4*	CCTTTGGCTCATGTGCTGAACT	CAGCGGCCATAAGTGTGGGTTT
*slc7a11*	TGGGTGGAACTGCTCGTAAT	AGGATGTAGCGTCCAAATGC
*gpx4*	GCAACCAGTTTGGGAGGCAG	CCTCCATGGGACCATAGCGC
*tfr1*	TCATGAGGGAAATCAATGATC	GCCCCAGAAGATATGTCGGAA
